# Fetal Teratomas: Advances in Diagnosis and Management

**DOI:** 10.3390/jcm13206245

**Published:** 2024-10-19

**Authors:** May Abiad, Nikan Zargarzadeh, Ali Javinani, Eyal Krispin, Alireza A. Shamshirsaz

**Affiliations:** Fetal Care and Surgery Center (FCSC), Division of Fetal Medicine and Surgery, Boston Children’s Hospital, Harvard Medical School, Boston, MA 02115, USA

**Keywords:** fetal teratomas, sacrococcygeal teratoma, fetal surgery, ex utero intrapartum treatment (EXIT)

## Abstract

Fetal teratomas, though rare, represent a significant proportion of tumors arising during fetal development. These tumors arise from pluripotent cells and can present in varying degrees of severity, ranging from incidental findings to life-threatening conditions. Prenatal imaging, via ultrasound and MRI, is necessary for diagnosis and risk assessment. The management of fetal teratomas, particularly those associated with complications like hydrops or airway obstruction, often requires a multidisciplinary approach. Interventions such as ex-utero intrapartum treatment (EXIT) procedures and minimally invasive alternatives have emerged as critical tools to improve neonatal outcomes in severe cases. Despite advances in fetal therapies, careful prenatal monitoring and individualized management remain essential, especially for tumors with high vascularity or those that risk compromising cardiac output. This review explores the diagnostic methods, management strategies, and outcomes associated with fetal teratomas, highlighting recent advancements that contribute to improving survival and reducing morbidity in affected neonates.

## 1. Introduction

**Figure 1:**
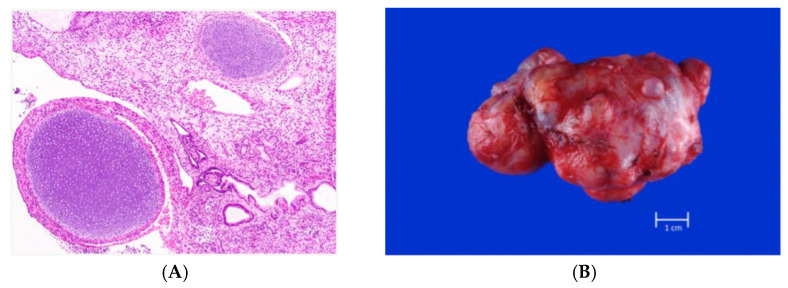
Immature teratoma, diagnosed in utero. (**A**) Histology. (**B**) Gross, post-resection.

Fetal teratomas, with an incidence ranging from 0.07 to 2.8 per 1000 pregnancies, account for most fetal tumors [1,2]. These tumors arise from pluripotent cells encompassing all three germ layers: endoderm, mesoderm, and ectoderm. While predominantly benign, fetal teratomas can be histologically classified into mature and immature types (Figure 1). Immature teratomas are distinguished by the presence of primitive neuroglial tissue and neuroepithelial rosettes [3,4]. These teratomas generally present with normal fetal karyotypes, although rare cases of chromosomal abnormalities have been reported in conjunction with certain teratoma types. The molecular pathogenesis of these tumors is not yet fully understood. The current evidence suggests that abnormal migration and differentiation of primordial cells during early development are involved in tumor formation.

Typically developing along the body’s midline, these tumors most frequently occur in the sacrococcygeal region, accounting for 60–80% of cases [5,6]. Other common locations include the head and neck, mediastinum, and less frequently, pericardial, intracranial, and retroperitoneal areas. Teratomas can present differently during the fetal period, ranging from incidental findings on antenatal imaging to severely affected fetuses and complicated pregnancies. Prenatal ultrasound demonstrates a high sensitivity in identifying fetal teratomas, revealing large irregular masses characterized by both solid and cystic components interspersed with calcified spots. Although there is no treatment available to directly target teratomas, advances in prenatal diagnosis and fetal surgery facilitate a more effective treatment of complications secondary to teratomas. These innovative fetal therapies have significantly improved long-term outcomes in affected neonates, offering hope for a better quality of life and reduced morbidity. The purpose of this narrative review is to provide a comprehensive overview of the diagnosis, management, and outcomes associated with fetal teratomas, demonstrating the evolving role of prenatal interventions in optimizing neonatal outcomes.

## 2. Sacrococcygeal Teratomas

### 2.1. Introduction

Sacrococcygeal teratomas (SCTs) are the most common congenital tumors in newborns, occurring in approximately 1 in 27,000 to 40,000 live births [7,8]. These tumors exhibit a marked female predominance, with a reported ratio of 3:1 or 4:1. SCTs can present either as a tumor of infancy or in utero, with some cases detected as early as 13 weeks of gestation [9]. The most common histological subtype prenatally diagnosed is a mature SCT [10]. The Altman classification system categorizes SCTs based on the location of the tumor [11]:

- Type I: Predominantly external with minimal internal components.
- Type II: Approximately equal external and internal components.
- Type III: Predominantly internal (pelvic) with some external presence.
- Type IV: Entirely internal.

**Figure 2:**
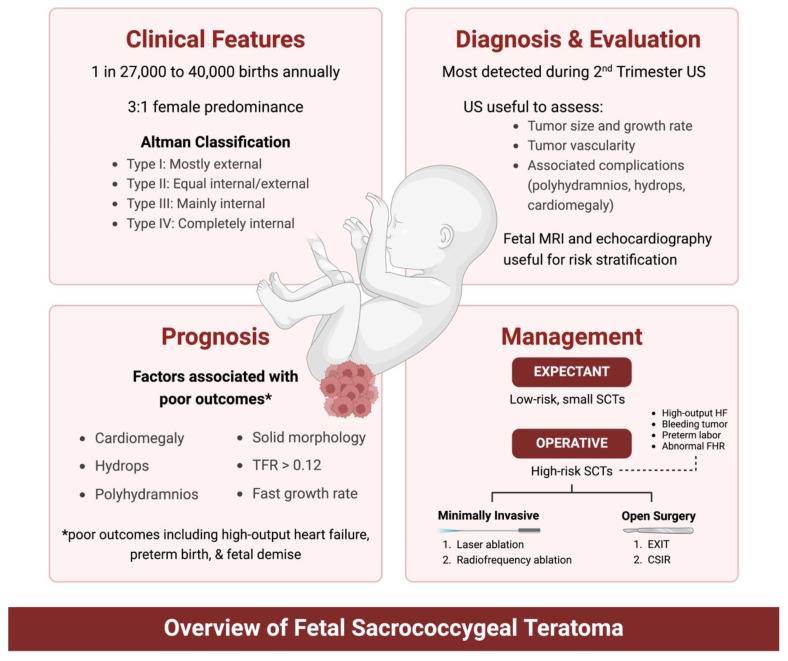
Overview of diagnosis and management of fetal SCT. US: ultrasound; MRI: magnetic resonance imaging; TFR: tumor-volume-to-fetal-weight ratio; SCT: sacrococcygeal teratoma; HF: heart failure; FHR: fetal heart rate; EXIT: ex utero intrapartum treatment; CSIR: cesarean section to immediate resection.

A large cohort study involving 37 SCT cases demonstrated that Altman Type I was the most common, accounting for 62% of cases, followed by Type III at 19%. Type IV SCTs are the least common (6%), often presenting with malignant elements and detected later in infancy [12]. In addition to the Altman classification, the Gonzalez-Crussi classification system further categorizes teratomas based on the degree of immaturity, from mature teratomas (Grade 0) to those with significant immature neuroectodermal tissue (Grades 2–3) [13]. This histological classification is particularly useful in assessing the tumor’s malignant potential and guiding postnatal management. Figure 2 provides an overview of the diagnosis and management of fetal SCTs.

### 2.2. Diagnosis and Evaluation

The prenatal diagnosis of SCTs most commonly occurs during the second trimester anatomical scan, although some cases have been identified in the first trimester [9,14,15,16]. Most SCTs diagnosed in utero are classified as Altman Type I or II [15]. Ultrasound is crucial for assessing tumor characteristics and risk stratification, particularly for identifying high-risk findings such as tumor size, vascularity, and associated complications [17,18,19]. Due to the vascular nature of these tumors, three-dimensional Doppler ultrasound can also be of particular use [14,17].

Recently, fetal MRI has been employed to more accurately characterize SCT lesions, focusing on the extent of intrapelvic expansion and compression of adjacent organs [20,21,22]. A study of 11 patients with prenatally diagnosed SCTs found that MRI offers superior detection of tumor extension, colonic displacement, and associated complications compared to ultrasound, with strong overall agreement between the two modalities [23]. MRI is especially valuable when the ultrasound diagnosis is unclear [24,25]. Fetal echocardiography is essential for evaluating cardiac output and valve function, allowing for risk stratification and more informed management of fetuses with SCT [26]. 

The key differential diagnosis for a cystic sacral mass in a fetus is a distal neural tube defect, or a myelomeningocele [15]. This condition presents with posteriorly splayed spinal elements and a meningocele. In contrast, sacrococcygeal teratomas are always located near the coccyx and typically create a presacral mass. Fetal MRI is especially useful to differentiate these two pathologies [27].

### 2.3. Prognosis and Natural History

Perinatal mortality for prenatally diagnosed SCTs ranges from 25% to 43% [15,26]. SCTs are often hypervascular and rapidly growing, leading to high-output cardiac failure due to a vascular steal phenomenon [28]. This can result in polyhydramnios, hydrops, and fetal demise. Notably, fetuses with an SCT and hydrops face a 2.7-fold increased risk of poor outcomes compared to those without hydrops [29]. Hemorrhagic complications are the most common cause of neonatal mortality in patients with SCTs [30,31]. Additionally, a rare but potentially life-threatening complication of an SCT with hydrops is maternal mirror syndrome (MMS) [32]. MMS is characterized by maternal symptoms mirroring fetal distress, including edema in the fetus and placenta, which can cause swelling in the mother such as scalp edema, pleural effusion, pericardial effusion, and ascites.

Several prognostic factors have been identified in patients with SCTs diagnosed prenatally [33,34]. In a study on 84 fetuses with diagnosed SCT, van Heurn et al. found that cardiomegaly and fetal hydrops were predictors of poor outcomes in these patients [26]. The tumor-volume-to-fetal-weight ratio (TFR) has also been described as an early prognostic marker in fetuses with SCTs. TFR is calculated using an ellipsoid formula and by comparing the greatest diameter of the external tumor in multiple dimensions by the estimated fetal weight, as determined by the Hadlock formula [35]. In particular, a TFR > 0.12 prior to 24 weeks has been associated with poor outcome in these patients [36,37,38]. Rodriguez et al. found that a TFR > 0.12 predicted the development of fetal hydrops and overall poor outcomes with 100% sensitivity and 83% specificity [37]. Recently, hepatomegaly, and abnormal ductus venosus (DV) Doppler measurements have also been described as highly predictive of perinatal mortality in fetuses with SCTs [39]. 

A 2023 meta-analysis of 12 studies involving 447 prenatally diagnosed SCTs confirmed the associations of cardiomegaly, hypervascularity, solid tumor morphology, fetal hydrops, placentomegaly, and TFR > 0.12 with poor outcomes, including prenatal or neonatal death [40]. Solid tumor morphology showed the highest risk (20-fold increase in poor outcomes). Additionally, fetal cardiomegaly and increased preload indices of the fetal venous system, such as the ductus venosus pulsatility index (DV-PI), were associated with maternal complications. The SCT growth rate, determined by the difference between tumor volumes on late- and early-gestation ultrasounds divided by the time interval, was shown by Coleman et al. to be an effective prognostic factor for adverse outcomes, such as high-output heart failure, preterm birth, and fetal demise [41]. Additionally, the risk of mortality from these complications was found to be proportional to the tumor volume growth rate. 

### 2.4. Management

Consequently, a comprehensive surveillance plan for pregnancies complicated by SCTs should be implemented [15,42]. This plan should include serial ultrasounds to monitor tumor size, amniotic fluid volume, and placental thickness. A Doppler ultrasound of the solid tumor portions is crucial for assessing blood flow and detecting any abnormal vascular patterns. Finally, fetal echocardiography, paired with Doppler measurements of fetal vessels, can be used to evaluate fetuses with a poor prognosis and accurately detect a high-output cardiac state before the onset of hydrops.

An important consideration for fetuses with SCTs is the risk of fetal anemia. The increased cardiac output required to supply the highly vascularized tumor can impose a significant hemodynamic burden on the fetus. This increased demand, when coupled with even mild anemia, can exacerbate the strain on the fetal heart, potentially leading to hydrops and fetal demise. The tumor’s hypervascular nature and the potential for hemorrhage can lead to anemia in the fetus [19]. Moderate to severe fetal anemia is suspected when Doppler ultrasound detects a middle cerebral artery peak systolic velocity (MCA-PSV) of ≥1.5 multiples of the median (MoM) [43]. To confirm the diagnosis, percutaneous umbilical blood sampling should be performed under ultrasound guidance. In cases of severe fetal anemia, intrauterine transfusion (IUT) is the preferred treatment [44,45]. This involves transfusing packed red blood cells directly into the umbilical vein under continuous ultrasound guidance, which helps to stabilize the fetal condition by boosting the hematocrit and improving oxygen delivery. Additionally, some clinicians advocate for the use of IUT with not only red blood cells but also platelets to manage these cases more effectively. This approach aims to address coagulopathy that may arise secondary to the SCT’s vascular nature. Furthermore, targeting the feeding vessels with coagulating factors can be considered to reduce blood flow to the tumor and minimize the risk of hemorrhage, thereby mitigating the anemic and hydrops-related complications.

**Figure 3:**
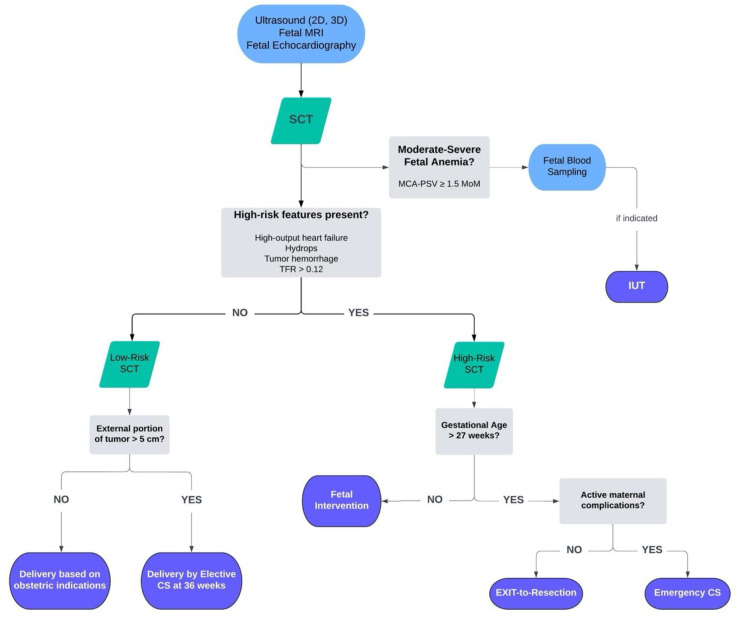
Algorithm for the management of fetal SCTs. MRI: magnetic resonance imaging; SCT: sacrococcygeal teratoma; TFR: tumor-volume-to-fetal-weight ratio; MCA-PSV: middle cerebral artery—peak systolic velocity; IUT: intrauterine transfusion; CS: cesarean section.

As SCTs can vary greatly, a tailored approach is essential. Figure 3 provides an algorithm for the management of fetal SCTs. For patients with isolated small SCTs and no additional concerning findings, expectant management with elective cesarean delivery after 36 weeks of gestation is often preferred to minimize the risk of complications, such as tumor rupture or obstructed labor [46]. Expectant management with vaginal delivery is a viable option in select cases of small tumor size [47]. However, for higher-risk SCTs, management strategies are categorized into minimally invasive procedures and open surgical interventions. Minimally invasive techniques, including in utero fetoscopic laser ablation of the dominant artery supply or radiofrequency ablation (RFA) of the tumor bed, are employed to reduce tumor size and mitigate complications while preserving fetal health [48,49,50]. For more advanced cases or when minimally invasive options are insufficient, open surgical approaches are utilized for tumor resection or open fetal debulking. Both approaches aim to reduce the strain of the growing lesion on the fetal cardiovascular system. 

#### 2.4.1. Minimally Invasive Approach

In a 2014 systematic review of 32 cases of SCT managed prenatally with minimally invasive surgery and 12 cases managed with open surgery, Van Mieghem et al. reported overall perinatal survival rates of 44% and 50%, respectively [49]. The survival rate was notably higher (67%) for fetuses undergoing minimally invasive procedures who did not have obvious hydrops. Additionally, the vascular approach to RFA has been shown to be more effective than the ‘interstitial’ ablation of the tumor in resolving hydrops in this population [50]. Minimally invasive techniques can also facilitate the performance of a low-segment uterine incision during cesarean delivery, as opposed to the classical incision required with open surgery. However, in another retrospective cohort that included eight SCTs managed with RFA, only one tumor showed signs of regression [51]. Additionally, this approach is not without risks, including thermal spread from ablation that can harm healthy tissue and lead to increased bleeding in the necrotic tumor bed [52]. 

#### 2.4.2. Open Approach

Open surgery is typically reserved for high-risk SCTs presenting with high-output heart failure, tumor hemorrhage, non-reassuring fetal heart patterns, or a risk for preterm labor [53]. This approach is contraindicated in cases of Type III or IV tumors, severe placentomegaly, or significant cervical shortening. Open surgery is most beneficial for fetuses with high-risk SCTs and hydrops, particularly when they develop at a gestational age that is too early for optimal neonatal care. In a case series of nine fetuses with SCTs managed at a tertiary referral center, Roybal et al. found the effectiveness of surgical intervention for high-risk SCTs to significantly decrease after 27 weeks of gestation, and proposed a treatment algorithm that emphasizes early delivery and immediate resection, which has been supported by follow-up studies [54,55]. For cases with rapidly progressive conditions before 28 weeks, immediate fetal intervention is recommended. This can include an EXIT-to-resection procedure, which involves partial delivery of the fetus while maintaining placental support for immediate tumor resection, or, if not feasible, postnatal resection. Additionally, the Cesarean-Section-to-Immediate-Resection (CSIR) approach, proposed by Creden et al., has shown promise [53]. In their retrospective review of 20 SCT cases, CSIR resulted in the survival of all three high-risk cases, with a median operative time of 156 min, thus making it an effective option for managing SCTs with signs of hydrops, fetal distress, or anemia.

In a recently published systematic review of 157 cases with prenatally diagnosed SCT, the survival rate was 56.2% for open fetal surgery, 45.8% for percutaneous interventions, and 71.0% for non-intervention cases, with no significant difference in survival between the operative groups [56]. However, the patients who underwent surgical management had more severe complications, including higher rates of hydrops, heart failure, and larger tumor sizes, which may explain the differences in survival outcomes. Additionally, both polyhydramnios and an earlier gestational age at delivery were more prevalent among non-survivors, thus showing the need for close monitoring of these patients. 

The long-term consequences of an SCT include tumor recurrence and ongoing issues with bowel and bladder control, particularly in those with a higher Altman classification and prenatal imaging suggestive of obstruction [51,57,58,59]. To monitor and manage these potential complications, follow-up care is essential for 3 to 5 years after treatment. This includes regular clinical examinations every three to six months, monthly tumor marker measurements such as alpha-fetoprotein and lactate dehydrogenase, and periodic imaging of the primary site along with chest X-rays. These measures help ensure timely detection of any recurrence or functional issues, supporting ongoing patient health and management.

## 3. Head and Neck Teratomas

### 3.1. Introduction

**Figure 4:**
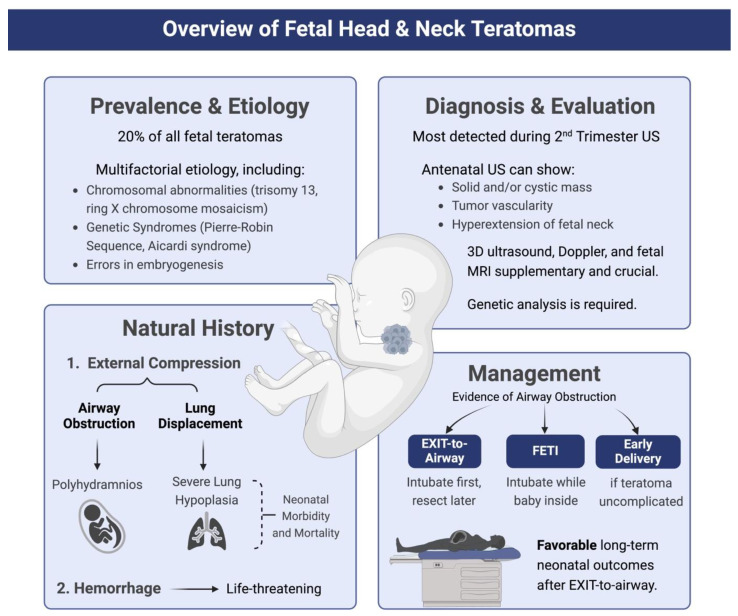
Overview for diagnosis and management of fetal head and neck teratomas. US: ultrasound; EXIT: ex utero intrapartum treatment; FETI: fetal endoscopic tracheal intubation.

Head and neck teratomas are reported to account for 20% of all prenatally diagnosed teratomas, and 30% of fetal tumors of the head and neck [6,60]. The pathogenesis involves the abnormal migration of primordial cells, which may settle in the mediastinum or hypothalamic regions [61]. Most head and neck teratomas are benign, but malignant forms have been reported, though they are exceedingly rare [62]. Their multifactorial etiology can include chromosomal abnormalities (such as trisomy 13, gonosomal pentasomy 49, XXXY karyotype), genetic syndromes (including Aicardi syndrome and Pierre–Robin sequence), and abnormalities in early embryonic development [63,64,65,66,67]. Given the complexity and potential for associated abnormalities, comprehensive evaluation and close monitoring are essential. Figure 4 provides an overview of the diagnosis, evaluation, and management of fetal head and neck teratomas. 

### 3.2. Diagnosis and Evaluation

**Figure 5:**
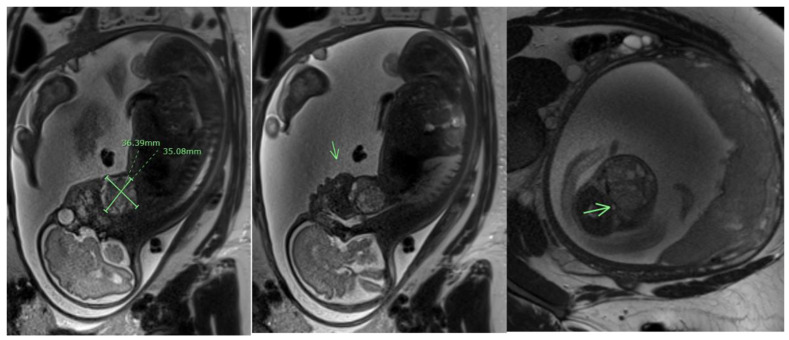
Fetal MRI of a cervical teratoma at 29 weeks and 1 day of gestation. Arrows point to the cervical teratoma.

Similarly to SCTs, most head and neck teratomas are detected during routine sonography in the late second and third trimesters [68,69]. A comprehensive prenatal evaluation is essential for prenatal counseling and management of these patients. On ultrasound, these teratomas typically present as an anterior or bidirectional facial or cervical mass, which may be partially solid or cystic [69,70]. The mass can protrude and cause the hyperextension of the fetal head, with severe perinatal sequelae if left untreated. Three-dimensional ultrasound has proven useful in enhancing prenatal diagnosis and aiding in the delivery planning of these patients [71]. Additionally, fetal MRI (Figure 5) enhances the diagnostic accuracy of the prenatal diagnosis, providing important information regarding the anatomy of the airway [72,73]. Prenatal imaging is also necessary in ruling out associated structural anomalies that may direct to the etiology. Finally, genetic analysis is required for all prenatally diagnosed teratomas to properly identify associated congenital abnormalities [6,74,75]. 

There are several important differential diagnoses of fetal head and neck teratomas. Lymphatic malformations typically present as cystic, less vascular masses compared to the solid or mixed composition of teratomas [76]. Another important differential is congenital cervical neuroblastomas, which tend to be located in the posterior neck region, unlike cervical teratomas that usually appear in the anterior or midline [77]. These conditions can be further distinguished using imaging modalities including ultrasound and MRI [78].

### 3.3. Prognosis and Natural History

Fetal head and neck teratomas can lead to serious complications due to their size and location. The external compression exerted by these tumors often leads to significant airway obstruction, impairing the fetus’s ability to swallow and resulting in polyhydramnios [74,79]. This obstruction can also force the fetal lungs upward, leading to severe lung hypoplasia, which can contribute to neonatal morbidity [80,81]. Teratomas may cause airway obstruction more frequently relative to other cervical masses such as lymphatic malformations [82]. Lymphangiomas typically have a more homogenous cystic structure, are less likely to invade adjacent tissues, and are often less vascularized. In contrast, teratomas are solid or mixed lesions, often with vascular components. The highly vascularized nature of these teratomas additionally poses a risk of substantial blood loss, further complicating the clinical picture [83]. The combination of airway compromise, impaired lung development, and potential for hemorrhage necessitates careful prenatal monitoring and planning to manage these risks and improve outcomes. 

The Tracheoesophageal Displacement Index (TEDI) is used to assess and quantify airway obstruction based on fetal MRI imaging. It is calculated by measuring the sum of the lateral (L) and ventral (V) displacements (in millimeters) of the tracheoesophageal complex from its normal anatomical location at the ventral aspect of the cervical spine. According to Lazar et al., factors such as giant neck masses, teratoma diagnosis, polyhydramnios, and a TEDI value > 12 mm accurately predict complicated airways, making ex utero intrapartum treatment (EXIT) a critical intervention for such cases [84].

### 3.4. Management

**Figure 6:**
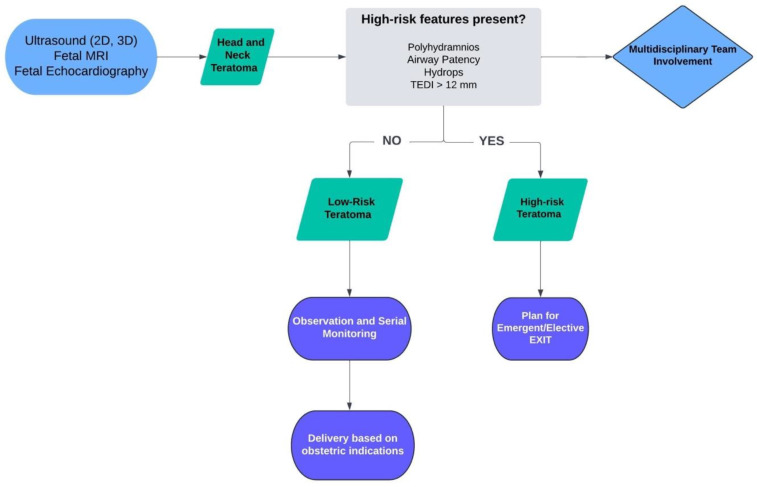
Algorithm for the management of fetal head and neck teratomas. MRI: magnetic resonance imaging; TEDI: tracheoesophageal displacement index; EXIT: ex utero intrapartum treatment.

The management of fetal head and neck teratomas differs from SCTs, as it requires specific considerations of fetal airway obstruction. Figure 6 provides an algorithm for the management of prenatally diagnosed head and neck teratomas. For small non-obstructing fetal neck masses, expectant management is often the preferred approach. In these cases, careful monitoring through prenatal imaging is essential to assess for any signs of airway compromise [85]. If the mass does not significantly impact the fetal airway, delivery can proceed without immediate intervention, followed by postnatal evaluation. After birth, a thorough assessment of the neonate’s airway and overall condition will guide the timing and approach for surgical resection of the mass [86]. Postnatal resection is typically performed once the neonate is stable and, depending on the size and complexity of the mass, may involve coordination with pediatric surgeons and specialists [83]. Postnatal surveillance includes repeated imaging and alpha-fetoprotein (AFP) measurements, as continued elevation in AFP levels can indicate metastasis or recurrence. 

Large congenital neck masses can cause significant airway compromise, leading to potential neonatal death if not appropriately managed. To address these issues, a range of fetal interventions can be employed, including EXIT-to-airway and fetal endoscopic tracheal intubation (FETI). The formation of a multidisciplinary team is essential to the survival of the neonate regardless of the operative approach.

#### 3.4.1. EXIT-to-Airway

EXIT-to-airway is a key intervention for fetuses with compromised airways due to neck masses [87]. This procedure involves the partial delivery of the fetus while maintaining placental support, specifically to facilitate intubation rather than immediate resection, as seen in SCT management. Patients undergoing EXIT are often close to or at term, with a reported mean gestational age of 36–37 weeks [77]. Originally developed to manage tracheal clips used in fetal tracheal occlusion therapy for congenital diaphragmatic hernia, EXIT-to-airway is effective for maintaining fetal airway patency and is particularly useful when a large neck mass or other complications are present [88]. In a study of 45 EXIT procedures at a large tertiary referral center, 35.6% were emergency cases, with a median maternal estimated blood loss of 800 mL and 13.3% of patients requiring blood transfusions [88]. Notably, 11 of these procedures were for cervical or upper airway teratomas, with 91% having associated polyhydramnios, 18% developing nonimmune hydrops, and 72% delivering preterm. The neonatal mortality rate was 18%, with 33% of survivors requiring a tracheostomy. 

Therefore, EXIT-to-airway has been established as a highly effective option for delivering babies with occlusive upper airway masses including teratomas, with survival rates reaching 82%. Recently, diagnostic fetoscopy has been offered prior to proceeding with the procedure to confirm airway patency, as ultrasound and MRI alone may not fully assess airway obstruction [89]. This approach can prevent unnecessary EXIT procedures and facilitate better management of affected fetuses. One potential downside to the EXIT procedure involves the need for general anesthesia and the use of inhaled anesthetics, associated with maternal complications such as persistent uterine atony and adverse effects on fetal cardiac function. EXIT provides ample time for airway control through intubation, tracheotomy, or tumor resection on placental support, ensuring comprehensive care for these complex cases.

The long-term outcomes of patients who have undergone EXIT-to-airway have been favorable [88,90]. In a Belgian cohort of 11 fetuses with prenatal upper airway obstruction, patients were followed up several years after birth [90]. Prenatal imaging and fetoscopic evaluation avoided EXIT procedures in eight cases by confirming accessible airways. For the remaining three patients, EXIT-to-airway procedures were performed, including tracheostomy and tumor resection. The long-term follow-up showed favorable outcomes for all patients, demonstrating that a combination of prenatal imaging, fetoscopy, EXIT, and neonatal surgery can optimize the long-term results and reduce the need for EXIT procedures. Speech difficulties have been reported as the most common long-term complication (88.2%) in babies with prenatally diagnosed oropharyngeal masses who underwent an EXIT procedure [91]. 

#### 3.4.2. Fetal Endoscopic Tracheal Intubation (FETI)

An emerging alternative to EXIT-to-airway, FETI involves securing the fetal airway via percutaneous endoscopic tracheoscopy under laryngoscopy and ultrasound guidance [92]. A study evaluating 35 fetuses with neck masses and suspected airway obstruction found that FETI was successfully performed in 8 out of 12 cases with confirmed obstruction, avoiding the need for EXIT [93]. FETI was chosen over EXIT-to-airway because it is a less invasive procedure that reduces maternal risks such as blood loss, wound infections, and potential uterine damage, which can affect future fertility. Unlike EXIT, which requires general anesthesia and complex uterine manipulation, FETI can be performed under epidural anesthesia, making it a safer option in many cases. Despite no reported maternal complications and successful intubation at birth, three neonatal deaths occurred due to postnatal complications. Further research is required to demonstrate the safety and efficacy of this approach.

## 4. Other Teratomas

### 4.1. Pericardial Teratoma

Pericardial teratomas are rare but severe fetal tumors, accounting for 9.5% to 19% of all primary cardiac tumors detected in utero [94]. They invariably present with an associated pericardial effusion, which can lead to cardiac tamponade and severe fetal hydrops, significantly worsening prognosis [6]. The prevalence of pericardial teratomas shows a slight male predominance, with a male-to-female ratio of approximately 1.3:1 [95]. A diagnosis is made through detailed ultrasound and fetal echocardiography [5].

Despite being histologically benign, pericardial teratomas can have a severe course. Their rapid growth may result in a mass effect on the heart and major thoracic vessels, causing severe fetal hydrops and, in some cases, intrauterine fetal demise. A study by Rychik et al. highlighted that tumor growth in cases of intrapericardial teratomas is extremely rapid and can lead to progressive cardiac output decline and hydrops [96].

Several prenatal interventions have been employed to improve outcomes in affected fetuses. These include pericardiocentesis to aspirate pericardial effusion, thoracocentesis for pleural effusion, amnioreduction for polyhydramnios, placement of a pericardioamniotic shunt, laser ablation of the tumor, and open fetal surgery (including EXIT) for tumor resection. Such interventions are particularly beneficial for hydropic fetuses.

A systematic review of 67 cases of intrapericardial teratoma revealed that fetuses without hydrops generally have a favorable prognosis if they undergo postnatal tumor resection, with a 92% success rate [97]. In contrast, perinatal death occurred in 21 cases, with 90% being hydropic. EXIT-to-resection procedures can be very beneficial, as demonstrated by Rychik et al., who reported successful resection in fetuses delivered via EXIT at 31 weeks and through open fetal surgery at 24 weeks [96].

The management algorithm for pericardial teratomas is analogous to that for SCTs. Fetuses with pericardial teratomas who are not hydropic can typically wait for postnatal resection. In cases with hydrops, intervention is stratified by gestational age: those with hydrops before 28 weeks may benefit from fetal intervention, while those with hydrops at 28 weeks or later may be managed with either pericardiocentesis or early delivery with postnatal resection, depending on fetal lung maturity [98].

### 4.2. Intracranial Teratoma

Intracranial teratomas are extremely rare but represent the majority of fetal intracranial tumors [6]. They are typically diagnosed in the late second or third trimester and may often be associated with a lack of definitive ultrasound findings. Fetal MRI is a valuable tool in these cases, and detailed fetal neurosonography should be considered to aid in diagnosis [99]. Unfortunately, the prognosis for fetal intracranial teratomas is poor, with survival rates reported to be less than 10% [4]. The prognosis is further compromised when there is a significant extension of the tumor into adjacent brain structures. At present, there are no viable fetal interventions for prenatally diagnosed intracranial teratomas. 

## 5. Limitations

While this narrative review covers key advancements in the field of fetal surgery for the prenatal management of teratomas, it is important to note that it reflects selected studies and expert opinions. As new clinical techniques and research studies emerge, future reviews will need to incorporate those findings to keep pace with the rapid developments in fetal care.

## 6. Conclusions

The management of fetal teratomas, though challenging due to the complexity and variability of these tumors, has seen significant advancements that have improved neonatal outcomes. The prognosis for sacrococcygeal, head and neck, and pericardial teratomas can be poor, particularly when associated with hydrops or significant tumor growth. However, a multidisciplinary approach combined with fetal surgery presents a promising avenue for intervention. For intracranial teratomas, postnatal surgical resection remains the preferred treatment. Procedures such as intrauterine transfusion, ablation, EXIT-to-airway, and in utero resection, have demonstrated the potential to significantly enhance survival rates and reduce morbidity. The evolution of prenatal diagnostic techniques and surgical strategies has provided a robust framework for managing these rare but life-threatening conditions, offering hope for better outcomes in affected fetuses.
